# High-resolution biophysical analysis of the dynamics of nucleosome formation

**DOI:** 10.1038/srep27337

**Published:** 2016-06-06

**Authors:** Akiko Hatakeyama, Brigitte Hartmann, Andrew Travers, Claude Nogues, Malcolm Buckle

**Affiliations:** 1 LBPA, IDA, ENS Cachan, CNRS, Université Paris-Saclay, F-94235, Cachan, France; 2 MRC Laboratory of Molecular Biology, Francis Crick Avenue, Cambridge Biomedical Campus Cambridge, CB2 0QH, UK; 3 Department of Biochemistry, University of Cambridge, Tennis Court Road, Cambridge, CB2 1GA, UK

## Abstract

We describe a biophysical approach that enables changes in the structure of DNA to be followed during nucleosome formation in *in vitro* reconstitution with either the canonical “Widom” sequence or a judiciously mutated sequence. The rapid non-perturbing photochemical analysis presented here provides ‘snapshots’ of the DNA configuration at any given moment in time during nucleosome formation under a very broad range of reaction conditions. Changes in DNA photochemical reactivity upon protein binding are interpreted as being mainly induced by alterations in individual base pair roll angles. The results strengthen the importance of the role of an initial (H3/H4)_2_ histone tetramer-DNA interaction and highlight the modulation of this early event by the DNA sequence. (H3/H4)_2_ binding precedes and dictates subsequent H2A/H2B-DNA interactions, which are less affected by the DNA sequence, leading to the final octameric nucleosome. Overall, our results provide a novel, exciting way to investigate those biophysical properties of DNA that constitute a crucial component in nucleosome formation and stabilization.

Gene expression is essentially controlled through the spatial and temporal distribution of nucleosomes on the genome. The presence of a nucleosome generally has an inhibitory effect on DNA binding proteins; indeed the binding of TATA binding protein and the whole pol II transcription machinery requires the absence of nucleosomes. There is much debate concerning the differential contribution of various factors such as sequence, remodellers and transcription factors to nucleosome positioning^1^,^2^ although in essence the central question concerns those molecular mechanisms that are involved in nucleosome formation, stabilisation and destabilisation.

Originally DNA condensation may have been initiated by wrapping of DNA around prototype histones using positioning parameters inherent in the DNA sequence. With the advent of the role of nucleosome shuffling mediated by chromatin remodellers in gene regulation the requirement for strong positioning signals may have been attenuated. A consequence of this idea is that the search *in vivo* for strong positioning sequences in modern genomes may be fruitless. However it is of considerable interest to try to determine the biophysical parameters of DNA that initiate nucleosome positioning and that probably served in primordial nucleosome binding.

Nucleosomes are formed by histone octamers consisting of two heterodimers H2A/H2B and one tetramer, (H3/H4)_2_ wrapping ~145/147 base pairs of DNA ~1.7 times around them in a left-handed supercoil with an average radius of curvature of 9 nm. The way in which a DNA sequence can intrinsically and specifically modulate its malleability and thus variations in the shape of the double-helix, is thought to be an essential factor in nucleosome formation^3^,^4^,^5^,^6^.

Ground breaking studies to identify SELEX-generated DNA sequences that possessed advantageous parameters for nucleosome formation^7^,^8^ lead to the suggestion of a positional code for nucleosome positioning and paved the way for crystallographic studies on reconstituted nucleosomes that provided remarkable insights in particular into the bound DNA shape^4^,^9^,^10^ and DNA-histone interface^11^.

Of note however, is that in none of these seminal works is the atomic structure of free DNA studied. Indeed, because of their length, the detailed structures of free 145/147bp DNAs cannot be revealed by traditional approaches such as X-ray diffraction or NMR. This hampered the comparison between free and bound DNAs, and thus the changes induced upon histone binding remained elusive as well as the mechanism of nucleosome formation and the exact nature of positional signals.

In this context, we have applied an approach developed in our group^12^ based on the measurement of the probability of UV induced cyclobutane dimer formation between adjacent pyrimidines (Y-Y dimer) on the same DNA chain^12^. This technique of photochemical analysis of structural transitions (PhAST), was applied to naked and bound DNA as a probe of changes in local base structure not only between naked DNA and reconstituted nucleosomes but also at different stages of nucleosome formation. UV induced Y-Y dimer formation has already been used to probe nucleosome core structure either by looking at the periodicity of photoproducts^13^ or by correlating the rate of Y-Y dimer formation with the degree of, and direction of, bending in nucleosomes^14^. The location of Y-Y dimers and intensities of photo-induced modifications are themselves affected by external agents that reshape the DNA structure and thus alter the photochemistry. However the incident UV light is in no way hampered by the presence of for example a protein. So it has to be borne in mind that although PhAST is not a footprinting technique, as for example in the case of DNase I or micrococcal nuclease, and thus does not give a precise idea of the contact area of a protein with the DNA, it provides unique information on the local DNA structure at a base level. We reveal photochemical products using a simple primer extension technique coupled to capillary electrophoresis. This confers high-resolution, excellent quantification, application *in vitro* and *in vivo*, and the possibility of high-throughput since practically any size DNA sample may be analysed in a fashion analogous to genome sequencing.

X-ray derived DNA structures containing a thymine-thymine dimer show that the formation of two C5-C5 and C6-C6 covalent bonds is characterized by marked positive rolls (~+20°, see Supplementary Fig. 1) and low twists (~25°)^15^. At a very simple level, the probability of inducing Y-Y dimer formation may be therefore modulated by the local architecture of naked DNA, in particular the roll and twist angles. The roll angle measures the rotation between two successive base-pair planes about their long axis (y-axis); the roll is positive when it opens up on the minor groove side of the bases, decreasing the distance between the two C5 or C6 atoms of successive pyrimidines. In both naked and bound DNA^16^,^17^ positive rolls are generally associated with low twist, thus minimizing the rotation between two successive base-pair planes about the z-axis and hence reinforcing the proximity between two successive bases. One would thus expect that maximal and minimal probabilities of Y-Y dimer formation are indicative of intrinsic positive roll/low twist and negative roll/high twist respectively, in the targeted DNA.

In the nucleosome, examination of the high-resolution X-ray structures confirmed that roll and twist are correlated^4^. Here, we chose to focus on the roll parameter to interpret the measured probabilities of Y-Y dimer formation in free and nucleosomal DNA. Indeed, this parameter is the major player accounting for DNA curvature in the nucleosome^4^. In addition, roll values show a spectacular periodicity along the nucleosome DNA, clearly less accentuated in the case of twist^9^. Of course, if no change in Y-Y dimer formation is observed this does not necessarily mean that the roll angles are not affected, Y-Y dimer formation could also be dependent on other factors; in fact, alterations in local flexibility will also affect the time-averaged probability of trapping a suitable Y-Y structure for Y-Y dimer formation. However, on the whole, for reasons that will be discussed in more detail below we interpret changes in Y-Y dimer formation as being indicative of alterations in roll angles. Accordingly, a decrease in probabilities of Y-Y dimer formation can be produced for three roll angle couples of free/bound DNA: i) positive to negative rolls ii) positive to less positive rolls and iii) negative to more negative rolls. An increase in probability of Y-Y dimer formation also relates to three roll angle couples, substituting negative rolls by positive rolls, negative to less negative rolls and positive to more positive rolls.

In an attempt to understand *ab initio* nucleosome formation at a given sequence from a dynamic point of view, we follow structural changes occurring at the base pair level in DNA, as nucleosomes are formed *in vitro* under decreasing ionic strength conditions. Despite the lack of corresponding DNA sequences *in vivo,* the possibilities of some bias in the selection process, and the arguments against the existence *per se* of positioning sequences *in vivo* advanced above, we used the “Widom” 601 sequence with the idea that its high affinity for the histones is due to a concentration of intrinsic structural properties favouring nucleosome formation. In addition, we considered a mutant sequence (601.2.4) designed to attenuate the putative positioning sequences of the 601 sequence.

Our results provide the first dynamic analysis of nucleosome formation that indicates, in agreement with recent data on nucleosome unwrapping^18^, that sequence dependent intrinsic properties of DNA strongly impact on nucleosome stability and, more specifically, on the recruitment of (H3/H4)_2_ that is the first stage of nucleosome formation *in vitro.* This first stage, that serves as a point of nucleation of DNA bending and defines the dyad axis, is thus critical for determining nucleosome positioning subsequent to H2A/H2B recruitment. Moreover, we propose that hydrophobic interactions could play a non-negligible role in the initial recognition by (H3/H4)_2_. Ultimately, we discuss the existence of positioning sequences in modern genomes and of putative signals persisting from ancient mechanisms of DNA compaction, and which are maintained during the first stage of nucleosome formation.

## Results

### Reconstitution of nucleosomes

Linear DNA fragments containing the 601 sequence (Supplementary Fig. 2a) and a mutated sequence (Supplementary Fig. 2b) were reconstituted separately with full length octamers (H2A/H2B/H3/H4)_2_, H2A/H2B dimers and (H3/H4)_2_ tetramers as described in Material and Methods. The mutated 601.2.4 sequence contains changes at putative positioning regions in the 5′ half of the 601 sequence, of particular interest for nucleosome stability^19^,^20^,^21^. Putative positioning motifs were identified from analyses of nucleosomal sequences -comprising artificial sequences such as the 601 sequence-, and consist of alternating T:A rich segments, preferentially situated at inward facing narrow minor grooves, and of G:C or TG.CA rich segments, occurring preferentially at outward wide minor grooves (for reviews see)^22^,^23^ likely because the 2-amino group of guanine sterically hinders narrowing of the minor groove^24^. In the mutant 601.2.4, the positions of base changes were largely designed to hinder the extreme narrowing of the minor groove where it faces the histone octamer. AGC, GTG and AGC were thus introduced at SHLs −3.5, −5.5 and −6.5, respectively. For the same purpose, TTGAT at SHL −1.5 replaced TTAAA, which is deemed crucial for nucleosome stability^9^,^21^,^25^,^26^. Other changes (C->A at SHL −1.0, CA->AT at SHL −2.2/−2.1, CC->TT at SHL −6.3/−6.2) could potentially shift the phasing of sequences favouring narrowing of the minor groove.

Micrococcal nuclease (MNase) digestion of reconstituted objects and naked DNA followed by fluorescent end labelled primer extension and capillary electrophoresis (described in Material and Methods) gave profiles shown in Supplementary Fig. 3. Fragments were generated using Taq polymerase expansion of 5′ end labelled 6-FAM fluorescent primers and extension was terminated at the cleaved base. Fragment size determination was determined using capillary electrophoresis. Calibration with known size markers allowed single base resolution identification of cleavage sites and calculation of the relative intensity of peaks as a function of the sequence position and thus calculation of the frequency of cleavage at each base. For the histone octamer reconstitution the MNase footprint extended from ~−70 to +70 around the dyad axis (Supplementary Fig. 3(1)a). The MNase footprint of the 601 fragment reconstituted with (H3/H4)_2_ showed a shorter footprint from ~−30 to +40 with respect to the dyad axis (Supplementary Fig. 3(1)b) whereas reconstitution with H2A/H2B dimers produced no MNase footprint (Supplementary Fig. 3(1)c).

On reconstituted mutant 601 fragments (601.2.4) containing the histone octamer, MNase digestion produced footprints after reconstitution (Supplementary Fig. 3(2)) that although identically positioned were apparently weaker than those seen on 601 fragments (enlarged central footprint in Supplementary Fig. 4). This was equally true for MNase digestion of fragments reconstituted with (H3/H4)_2_. No footprints were observed for MNase digestion of mutant fragments reconstituted with the H2A/H2B dimers. The weaker footprints seen on the mutant fragment could be due to two reasons; either there is a mixed population of bound and unbound DNA or even if the majority of DNA is involved in a complex, these are more dynamic and therefore more accessible to MNase digestion. We believe that the latter is the case for arguments that will be developed later.

### UV Photochemical analysis of structural transition (PhAST)

**Figure 1:**
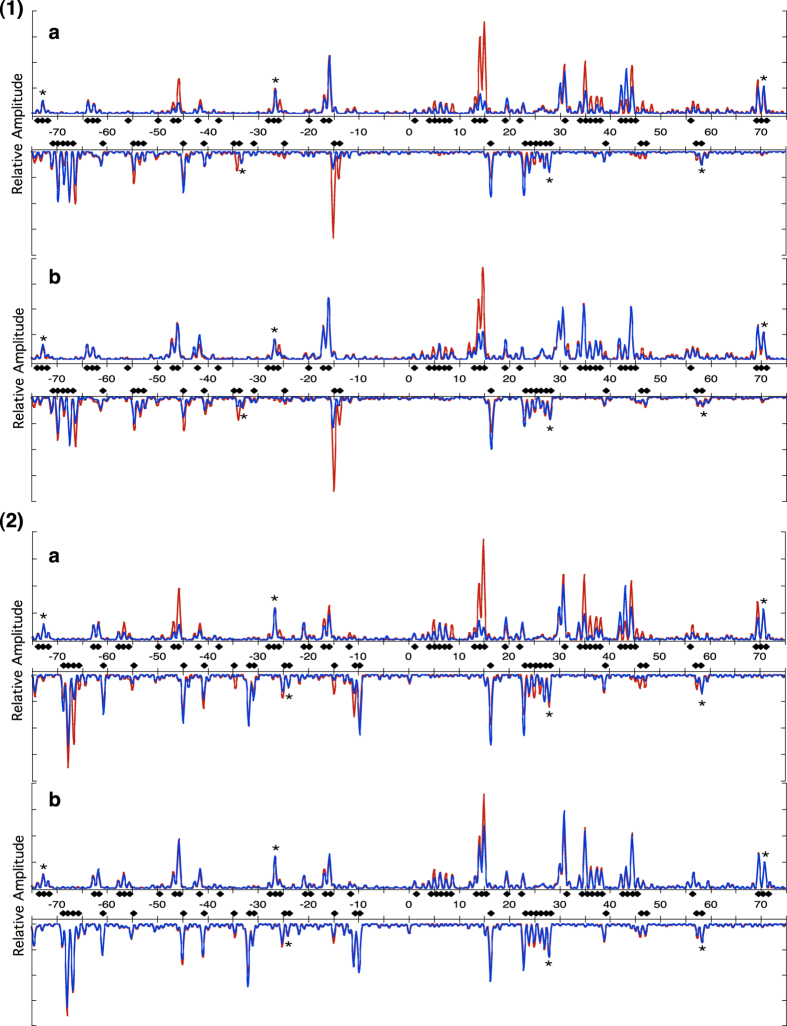
(**1**) Primer extension then capillary electrophoresis after photo-irradiation of 601 fragments (a) histone (H3/H4)_2_ tetramers and H2A/H2B dimers, (b) only (H3/H4)_2_ tetramers. (**2**) 601.2.4 fragments. (a) histone (H3/H4)_2_ tetramers and H2A/H2B dimers, (b) only (H3/H4)_2_ tetramers. Red lines show DNA alone, blue lines show reconstituted DNA. Peak size was normalized by reference peaks indicated by asterisks; the same set of peaks was used for all normalisation in all the photochemical change analysis. Closed diamonds indicate the positions of Y-Y steps on the 601 and 601.2.4 sequences.

Laser UV radiation of DNA alone or in reconstituted DNA was carried out as described in Material and Methods and following primer extension of fluorescent end labelled oligonucleotides separation of the ensuing fragments provided the patterns shown in Fig. 1.

On DNA alone, on both the 601 and the mutant sequences the overwhelming majority of termination points preceded the potential presence of a Y-Y dimer (Fig. 1(1) red curve and1(2) red curve). Photo-irradiation of DNA reconstituted with octamers showed an altered pattern of photo-reactivity (Fig. 1(1) blue curve and 1(2) blue curve). Changes in photo-reactivity occurred only on the 601 and mutant sequences and not beyond into the 5′ and 3′ extensions; the primers extended from −224 to 226 for the 601 sequence and −236 to 205 for the mutant, with respect to the dyad axis. However we did not examine extensively sequences across the whole plasmid outside of these boundaries, and it is indeed likely that nucleosomes are also being formed elsewhere. Where nucleosomes are shown to be present there are changes in photo-reactivity that do not occur in adjacent regions where no micrococcal nuclease footprint is observed, thus photochemical changes are reserved for those regions where a nucleosome has been formed. Peak size was normalised by reference to peaks within the footprint of the nucleosome that had not altered following reconstitution of nucleosomes compared to naked DNA as described in the legend to Fig. 1. We also compared changes using peaks outside of the footprint of the putative nucleosome that did not change after reconstitution and obtained exactly the same profiles (data not shown) so for clarity we used comparisons with unchanged peaks within the footprint. Dramatic changes in peak intensities occurred at many specific positions on the DNA sequence when reconstitution was carried out with DNA either in the presence of (H3/H4)_2_ tetramer and H2A/H2B dimers (Fig. 1(1)a and 1(2)a) or in the presence of (H3/H4)_2_ tetramer alone (Fig. 1(1)b and 1(2)b). No significant differences were seen when either DNA sequence was reconstituted with H2A/H2B dimers alone (data not shown). From the data presented in Fig. 1 and coherent with the data from the MNase footprinting (Supplementary Fig. 3) it is clear that the 601 and mutant sequences are involved in a nucleoprotein complex. In both cases there are very specific Y-Y steps that undergo significant differences in photo reactivity in nucleosomes compared to naked DNA. Particularly important are changes at positions −15 and +15 with respect to the dyad axis, which correspond to outsized photo reactivities of TT steps in naked DNA (Fig. 1). These steps belong to TTAAA elements that, in their free state, are characterized by atypical features, comprising a very narrow minor groove^20^ associated with marked positive rolls and low twists^27^. As pointed out in the Introduction, such a local structure promotes Y-Y dimer formation by decreasing the distance between the two C5 or C6 atoms. According to our analysis of the crystallographic structures of nucleosome containing TTAAA elements, the TT steps have average roll and twist of −4.5 ± 5° and 37.5 ± 3° respectively, which are much less favourable to the dimer formation. Thus, the enhanced changes obtained here at positions −15 and +15 are likely representative of an induced transition from a particularly photo-reactive structure towards a more refractive conformation.

**Figure 2:**
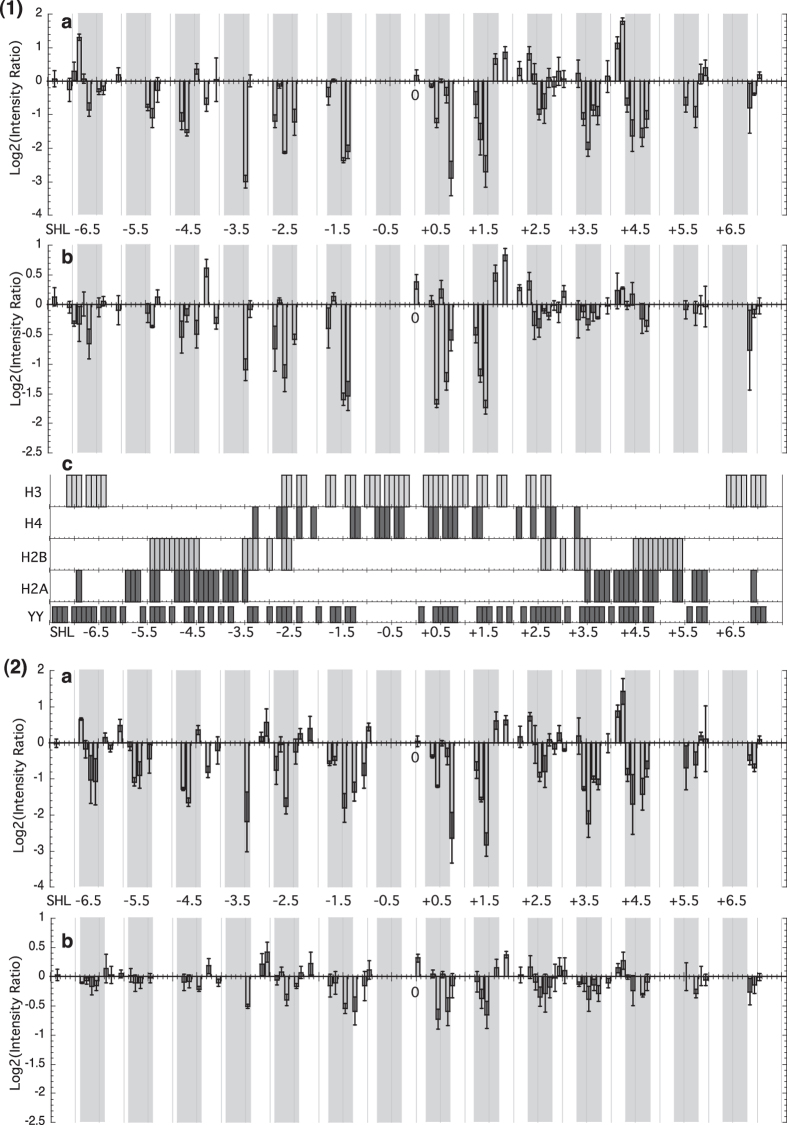
Log2 of the intensity ratios of the peak height for Y-Y dimer peaks for reconstituted DNA compared with naked DNA with (**1**) 601 fragments and (a) histone (H3/H4)_2_ tetramer and H2A/H2B dimers, (b) only (H3/H4)_2_ tetramer. Changes in the ratio at each base step on both strands are shown. As additional information, (c) shows the DNA residues involved in the interface with H3, H4, H2A and H2B, and, just below, the location of pyrimidine-pyrimidine (YY) steps. (**2**) 601.2.4 fragments and (a) histone (H3/H4)_2_ tetramer and H2A/H2B dimers, (b) only (H3/H4)_2_ tetramer. Note that the y-ranges of panels (a) and (b) differ. Minor-groove inward facing regions observed in the nucleosome crystal structures are represented by grey boxes.

In order to fully appreciate the differences and to escape the limitations imposed by the quantum yield of specific pyrimidine base steps, a presentation taking the log2 of the signal intensity ratios of each peak between reconstituted DNA and naked DNA as a function of super helical location (SHL, which refers to the periodic orientation of the DNA major and minor grooves with the histone core^28^. SHLs ±0.5, ±1.5, ±2.5 etc. indicate the locations where the DNA minor groove is oriented towards the histone core) was chosen to present the results, as shown in Fig. 2. In this representation Y-Y dimers occurring between Y_n_ and Y_n−1_ are taken as being at position SHL_n_. The advantage of this representation is that a more global appreciation of changes is seen rather than just the predominant specific changes seen for example at −15 and +15 with respect to the dyad axis shown in Fig. 1. As several PhAST experiments were performed on each of the different systems, a change was considered as significant, and discussed only, when the mean of the log2 quantity is superior to the corresponding standard deviation.

### Changes in the probability of Y-Y dimer formation upon octamer binding

The 601 and mutant sequences of 147bp inserted in plasmids differ by 15 bases all located in the 5′ half, spread from SHL-7 to SHL −1 (Supplementary Fig. 2). This difference led to a gain of 2 Y-Y steps in the mutant 601.2.4 sequence (72 Y-Y) compared to the 601 sequence (70 Y-Y) between SHL −7.3 and the centre (SHL 0). Furthermore the location of several Y-Y steps changes (Supplementary Fig. 2). Thus the mutant sequence gains 4 Y-Y steps between SHL −2.5 and SHL −0.5 compared to the 601 sequence (Supplementary Fig. 2).

In both sequences, 62% of the Y-Y steps, distributed along the whole DNA length, show significant changes in probabilities of Y-Y dimer formation between reconstituted and naked DNA (Fig. 2). Thus histones alter the structural characteristics of most naked YY steps along the whole occupied DNA sequence. Furthermore, taking into account the sequence differences, changes are identical in the 601 and mutant sequences, in terms of location and direction of the change i.e. an increase or decrease.

A comparison between the data of Fig. 2(1)a and 2(2)a shows that the amplitudes of the signals observed in the 5′ half of the mutant sequence from SHL −3.5 to SHL −1.5 decreased by up to 30% compared to the 601 sequence. One explanation for weaker MNase footprints (Supplementary Fig. 3) is that there is a mixed population of bound and unbound DNA. Were this the case, and bearing in mind that PhAST is not a footprinting technique but is reporting local changes in DNA structure then the distribution of photo reactive changes would be homogenous. This is not observed. The photo reactivity represents a (relatively rapid, of the order of ns) time-averaged representation of the whole population. These results suggest that in nucleosomes involving the mutant sequence, the left hand side of the dyad axis is less rigidly constrained structurally than in the 601 containing nucleosomes.

In accordance with the assumptions concerning the correlation between changes in roll angles and the probability of Y-Y dimer formation as explained in the introduction, an interpretation of the difference photo-reactivities shown in Fig. 2 ideally requires a comparison of the rolls in naked and reconstituted DNA. Because of their length, naked nucleosomal sequences cannot be studied by classical approaches providing atomic models, such as X-ray diffraction or NMR. Thus, the structures of naked 601 and mutant sequences are unknown. However, crystallographic structures of nucleosomes offer the opportunity to examine potential relationships between the rolls in bound DNA and the changes in probability of Y-Y dimer formation observed between naked DNA and DNA reconstituted with the histone octamer. Among the available structures of nucleosomes containing the 601 sequence, we chose to analyse those structures obtained at the highest resolution, namely, 3MVD (resolution 2.9 Å)^29^, 3LZ0 and 3LZ1 (resolution 2.5 Å)^25^, in order to limit uncertainties in particular regarding the side chains rotamers.

**Figure 3:**
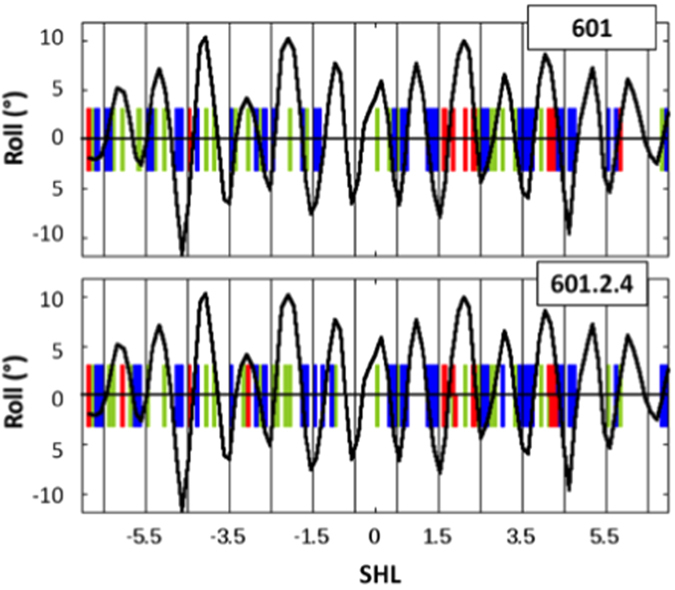
Roll angles in X-ray structures of nucleosomes and changes in probabilities of Y-Y dimer formation upon histone octamer binding. The periodic variations of roll values along the DNA in nucleosomes are represented with a black line, using a natural smoothing spline approximation. These roll values were calculated and averaged on three X-ray structures of nucleosomes containing the 601 sequence (PDB codes 3LZ0, 3LZ1 and 3MVD). The rolls of the pyrimidine-pyrimidine steps that correspond to decreases and increases in probability of Y-Y dimer formation obtained by comparing DNA bound to the histone octamer and naked DNA are represented by vertical blue and red bars, respectively. The remaining pyrimidine-pyrimidine steps for which no change was observed are positioned by vertical green bars.

Most decreases in probability of Y-Y dimer formation between naked DNA and reconstituted DNA of both 601 and mutant sequences correspond to negative roll regions in nucleosomes (Fig. 3). In the nucleosome, negative rolls occur in regions in which the minor groove faces and interacts with the histone core^9^; a reasonable assumption is that the structure of the dinucleotide steps involved in the interaction interface is stabilised and their thermal fluctuations reduced. Such constrained negative rolls could thus systematically disfavour Y-Y dimer formation. Positive roll regions, which interact much less with the histone core, are likely less constrained than the negative roll regions. This could explain why relatively few pyrimidine-pyrimidine steps with positive roll in nucleosomes correspond to an increase in probability of Y-Y dimer formation (Fig. 3).

In summary, our PhAST approach unambiguously reflects the DNA conformation in nucleosomes formed by both 601 and mutant sequences and also confirms that protein-DNA interactions tend to be weakened by the mutations introduced in the 5′ half of the 601 sequence^21^. The differences observed between the probabilities of Y-Y dimer formation in naked and reconstituted DNA reveal most SHLs of negative rolls in nucleosomes - apart from the SHL −0.5 region that does not contain a suitable Y-Y step. This relationship together with the large number of changes in the probability of Y-Y dimer formation suggests that the local structure and dynamics of naked 601 and mutant sequences are modified in the nucleosome, at least for Y-Y steps.

### Probability of Y-Y dimer formation upon (H3/H4)_2_ binding and the DNA-(H3/H4)_2_ interface

As for the histone octamer, the binding of (H3/H4)_2_ generates differences in probabilities of Y-Y dimer formation between naked and reconstituted 601 (Fig. 2(1)b and mutant 2(2)b) sequences. These differential photo-reactive patterns are expected to be due to (H3/H4)_2_ binding and thus, they were examined in relation to the DNA-protein interface in the crystallographic structures of nucleosome containing 601 sequence, 3MVD, 3LZ0 and 3LZ1 (see Supplementary data for methodological details). Note that this interface reports only those contacts between the DNA and the structured histone core; the histone tails not being resolved in the structures considered.

As shown in Fig. 2(1)c, the interface between the DNA and the structured part of (H3/H4)_2_ is concentrated in the central part of the DNA (from SHL−3.3 to SHL +3.3), with additional H3 contacts with the 5′ (from SHL − 7 to SHL −6.4) and 3′ (from SHL +6.4 to SHL +7) ends of the DNA.

The differential photo-reactivity pattern of 601 sequences with (H3/H4)_2_ (Fig. 2(1)b) compared to the octamer equivalents (Fig. 2(1)a) is quite revealing.

The number and the nature, decreases or increases, of the changes occurring in the central region contacted by (H3/H4)_2_ in the full nucleosome, from SHL −3.3 to SHL +3.3, are the same on the DNA reconstituted with the octamer or (H3/H4)_2_. The intensity of almost all the peaks in the reconstituted tetramer decreased compared to that for the reconstituted octamer. This could be due to lack of H2A/H2B interactions with the tetramer that would stabilise the complex; this will be discussed more in the light of data shown later. Nevertheless the photo-reactivity at specific positions was significantly different between the octamer and the tetramer. At SHL +0.7 the increased negative log2 intensity ratio of 0.4 for the octamer increases to 1.4 for the tetramer corresponding to a lower probability of Y-Y dimer formation at this position with the tetramer. At SHL +0.8 the negative intensity ratio decreased from 2.9 in the octamer to 0.6 in the tetramer. These two positions are where the most significant changes occur in this region but in fact SHLs from +0.5 to +0.8 are involved. These observations imply that this region in the H3/H4 tetramer complex is distorted differently from the same region in the octamer complex. Indeed, a careful examination of the X-ray structures of nucleosome reveals that a large part of the C-terminal domain of H2A bridges the α1 and α3 helices of H3 near the points where H3 contacts the DNA. This interaction between H2A and H3 could induce subtle changes in the orientation of H3 with respect to the DNA leading to a conformational DNA response different from that occurring in absence of H2A.

On the 5′ side of this central region, of particular interest are the series of significant changes, which are observed in the presence of H3/H4 but which are outside of the expected interface. Compared to the octamer differential photo-footprint, most of these signals are clearly weakened and indeed two of them are inverted at SHL −4.4 and −4.2. Additional inversions occur on the outermost 5′ half, around SHL −6.5. On the 3′ half of the 601 sequence, for SHL >+3.3, changes are also produced by H3/H4, with only one inversion at SHL + 3.4. Globally, these signals are however marginal compared to those observed on the 5′ half with the notable exception of a strong decrease just beyond SHL +6.5.

(H3/H4)_2_ bound to mutant sequences also provokes differential photo-reactivity, but the intensities of changes are very weak (Fig. 2(2)b). The most intense peaks are observed in the central region, as for the 601 sequence however their intensities remain largely inferior to those of both octamer-mutant (Fig. 2(2)a) and (H3/H4)_2_-601 (Fig. 2(1)b) complexes. A comparison of the differential photo-reactivities of the 601 (Fig. 2(1)b) and mutant (Fig. 2(2)b) sequences with (H3/H4)_2_ regardless of the differences in intensities of changes, shows that they are in fact very similar, suggesting common interaction events.

These observations strongly suggest that, even in the absence of H2A and H2B, the centre of the 601 sequence is a preferential site for binding of H3/H4. The presence of marked changes in the 5′ half of the sequence could designate this region as a secondary, less propitious (H3/H4)_2_ binding site. Another possibility is that, at the same time where the (H3/H4)_2_ is located in the central DNA part, the unbound 5′ region is involved in some form of interaction with H3. Indeed the large patch of positively charged amino acids of H3 that is engaged in contacts with the DNA ends in the native nucleosome could transitorily capture the DNA, inducing structural distortions even in the absence of H2A and H2B. Strikingly however, a comparison of photo-reactive change intensities in the 5′ and 3′ halves indicates asymmetric properties in the 601 sequences, with respect to the final dyad axis. Finally, the comparison of the effects of (H3/H4)_2_ reconstitution on the two DNA sequences demonstrates that the mutations in the 5′ half of the 601 sequence severely reduce the global ability of the mutant sequence to interact with (H3/H4)_2_.

### Probability of Y-Y dimer formation during the nucleosome reconstitution process

The whole process of nucleosome formation is a dynamic process. Although the experimental reconstitution requires a change in ionic strength that would apparently obstruct a kinetic analysis of this process, advantages of the PhAST technique are that it is independent of the reaction conditions (e.g. salt concentration) and is extremely rapid; the induced photochemistry occurs over a time scale (5 ns) that is too short to interfere with the DNA structure. The approach can thus be used to get ‘snapshots’ of DNA conformation during nucleosome formation. We therefore looked at the probability of Y-Y dimer formation as a function of ionic strength during the reconstitution process.

**Figure 4:**
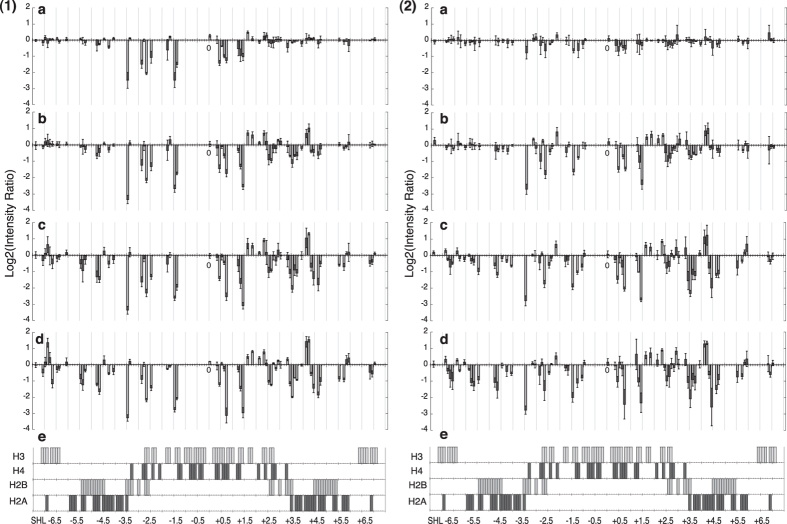
Snapshots during nucleosome reconstitution in (**1**) the 601 sequence and (**2**) the 601.2.4 sequence. The histograms represent Log2 of the intensity ratios of the peak height for Y-Y dimer peaks for reconstituted DNA with histone (H3/H4)_2_ tetramer and H2A/H2B dimer compared with naked DNA at (a) 1.5 M NaCl, (b) 1.0 M NaCl, (c) 0.5 M NaCl and (d) 0.25 M NaCl buffer during the salt dilution process. Error bars derive from SD of three separate experiments. (e) DNA regions involved in the interface with the structured part of each histone in the complete nucleosome.

PhAST was carried out on samples at each dilution step during the reconstitution process (Fig. 4) and again in order to correctly assess the progression of changes at each position, the log2 of the intensity ratios of the peak height for Y-Y dimer peaks at a specific ionic strength compared to the peak height of the DNA alone is shown as a function of SHL. This allows an appreciation of the evolution of signals through the changing conditions.

As seen in Fig. 4(1)a the photo-reactivity pattern of the 601 sequence at 1.5 M NaCl compared to naked DNA testifies to numerous changes. Marked effects are located between SHL −3.5 and SHL +2.5 (Fig. 4(1)a), commensurate with the formation of interactions between (H3/H4)_2_ and the central part of the 601 sequence. More specifically, the intensities of the signals on the 5′ side of the dyad (around SHLs −3.5 and −2.5) are similar to those observed with the octamer at low ionic strength (Fig. 2(1)a). This 5′ DNA part thus appears as a strong anchoring region for initialising nucleosome reconstitution. Additional weak but significant signals outside the centre likely correspond to the beginning of the recruitment of H2A and H2B (Fig. 4(1)a), it should be borne in mind that at 100 mM NaCl the (H3/H4)_2_:H2A/H2B dimer interface was shown to be not stable in the absence of DNA^30^ and that under these conditions H2A/H2B dimers transfer to (H3/H4)_2_ when these latter are bound to DNA^31^. Upon transfer to 1.0 M NaCl changes at SHL −4.5 appear more clearly in the region of H2A and H2B core binding (Fig. 4(1)b).

At the 3′ side of the dyad, changes from SHL +1.5 to SHL +4.5 are compatible with adjustments of H3/H4 and the expansion of H2A and H2B influence (Fig. 4(1)b). Changes at SHLs ±4.5 are particularly interesting because these regions are also contacted by the H2B N-terminal tail. According to the only X-ray nucleosome structure in which the histone tails were observed (PDB ID 1KX5^11^) H2B tails pass through the DNA gyres, contacting SHLs −2.5 and +4.5 and, symmetrically, SHLs +2.5 and −4.5 thus bridging the DNA gyres. Our data reinforces this image showing the events as they take place during reconstitution. In decreasing ionic strength conditions signals first appear at SHL −2.5 then at +4.5 and +2.5, +4.5 continues to evolve with the signal at −4.5 appearing.

At 0.5 M NaCl, the photo-reactivity pattern at SHL <−3.5 and SHL >+3.5 suggests the reinforced presence of H2A and H2B and, importantly, the involvement of the 5′ and 3′ DNA ends in the interface with H3 and H2A (Fig. 4(1)c). At this stage, all of the rearrangements along the whole sequence have taken place and their intensity suggests a large population of reconstituted nucleosomes. Indeed the only appreciable difference seen at 0.25 M NaCl is the intensity of the signals around SHLs −6.5, −5 and +6 attesting to completion of the superhelical pathway around the nucleosome.

The situation for the mutant sequence at 1.5 M NaCl differs remarkably from the 601 sequence. Very weak photochemical changes are observed (Fig. 4(2)a), the most significant being clustered in the vicinity of the dyad axis. This confirms that the mutations in the 5′ part of the centre of the 601 sequence are sufficient to disfavour the (H3/H4)_2_ interaction, as pointed out above by comparing the 601 and mutant sequences in the presence of (H3/H4)_2_ during reconstitution and at low ionic strength. From 1.0 M NaCl and concentrations below (Fig. 4(2)b–d) the evolution of differential photo-reactivity resembles that which is observed with the 601 sequence (Fig. 4(1)b–d), taking into account the differences between the two sequences. A curious point with the mutant sequence is the noticeable increase at SHL +6.9 at 1.5 M NaCl that becomes a decrease at 1 M NaCl and then almost disappears while clearly discernible at low ionic strength (Fig. 2(1)b). Within the limits of the accuracy of the photochemical probe, this could be interpreted as being due to an enhanced dynamics of the DNA 3′ end during nucleosome reconstitution.

These results provide a scenario to describe the succession of events during nucleosome reconstitution. With the 601 sequence, early events occur even at high salt (1.5 M NaCl), and involve in particular (H3/H4)_2_ binding to the DNA centre, to a greater extent on the 5′ side of the dyad axis. As the ionic strength is progressively dropped to 0.5 M NaCl, H2A/H2B docking occurs, first on the 3′ DNA side and then symmetrically with regard to the dyad axis. This H2A/H2B positioning is concomitant with the development of contacts involving the extreme ends of the 601 sequence, which are definitively stabilized at 0.25 M NaCl. With the mutant sequence, the first step is much more attenuated. Reconstitution really starts at 1 M NaCl, with the interaction of (H3/H4)_2_ at the central region of the DNA and one H2A/H2B dimer with the 3′ neighbouring region. Indeed, from 1.0 to 0.25 M NaCl, the succession of events remarkably parallels that which is observed with the 601 sequence. Consequently, mutation of the six bases between SHL −3.5 and SHL 0 creates a much more dramatic effect on reconstitution than mutation of the nine bases located between SHL −7 and SHL −3.5. This observation implies that the sequence of the central DNA part is crucial for optimizing nucleosome formation.

### Probability of Y-Y dimer formation during the (H3/H4)_2_ tetramer reconstitution process

**Figure 5:**
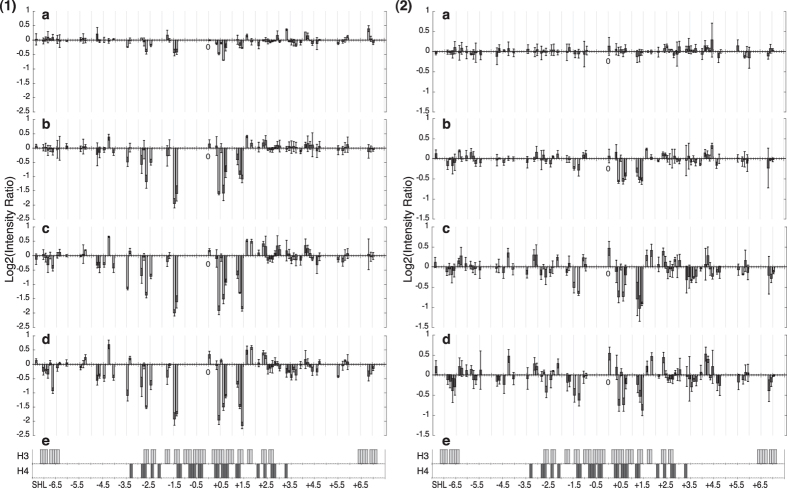
Snapshots during nucleosome reconstitution with histone (H3/H4)_2_ tetramer in (**1**) the 601 sequence and (**2**) the 601.2.4 sequence. The histogram represents log2 of the intensity ratios of the peak height for Y-Y dimer peaks at a specific ionic strength for reconstituted DNA compared with free DNA. (a) Peak height ratio of DNA alone to reconstituted DNA in 1.5 M (a), 1.0 M (b), 0.5 M (c) and 0.25 M (d) NaCl buffer during the salt dilution process. Error bars derive from SD of three separate experiments. (e) DNA regions involved in the interface with the structured part of each histone in the complete nucleosome. Note that the y-ranges of panels (**1**) and (**2**) differ.

A similar analysis was carried out for reconstitution with (H3/H4)_2_ and the results are shown in Fig. 5.

On the 601 sequence with (H3/H4)_2_ at 1.5 M NaCl (Fig. 5(1)a), the signals around the dyad axis are similar, albeit much less pronounced than with the octamer at the same ionic strength (Fig. 4(1)a). Between SHL −3.3 and SHL +3.3, the difference between the intensities of changes induced by H3/H4 (Fig. 5(1)a) and the octamer (Fig. 4(1)a) is in fact greater at 1.5 M than at low ionic strength (Fig. 2(1)b vs. 2(1)a). In addition to their global weakness, there is no asymmetric pattern between the 5′ and the 3′ sides around the dyad as seen with the octamer (Fig. 4(1)a). Furthermore, the pattern obtained with (H3/H4)_2_ at 1.5 M NaCl contains five inverted peaks (decrease ↔ increase at SHLs −4.2, −1.7, +5, +3.4 and +6.9) compared to those observed at 0.25 M NaCl (Fig. 5(1)d) or low ionic strength (Fig. 2(1)b). These inversions are not induced at the same ionic strength in the presence of the octamer (Fig. 4(1)a). Effects due to the presence of H2A/H2B on the DNA were weakly discernible during the first stage of octamer reconstitution (Fig. 4(1)a).

Indeed no direct effect of H2A/H2B on DNA was detected even at the lowest salt concentrations, the implication is that H2A/H2B interactions with the DNA necessitates the presence of the tetramer.

However, these results coupled to those seen in Fig. 5(1)a strengthen the idea that, at 1.5 M NaCl and in the presence of the four histone types, H2A and H2B help position H3/H4 at the DNA centre.

With decreasing salt (Fig. 5(1)b–d) the differential photo-footprinting patterns on the 601 sequence with (H3/H4)_2_ becomes more and more similar to those seen for (H3/H4)_2_ at low ionic strength (Fig. 2(1)b).

The situation on the mutant sequence with respect to the (H3/H4)_2_ tetramer reconstitution is shown in Fig. 5(2). The differential photo-reactivity pattern at 1.5 M NaCl does not contain any significant change (Fig. 5(2)a), unsurprisingly given the results presented in the above sections with regard to this sequence. At 1 M NaCl, several changes occur in the central region of DNA (Fig. 5(2)b). No significant signal is observed outside the centre, contrary to the pattern obtained at the same ionic strength and on the same sequence but in the presence of the four histone types. Of interest is that the most evident changes are located at the 3′ side of the dyad and this clearly shows that the 5′ side mutations strongly disfavour interactions with H3/H4. Indeed, the situation is stabilised only at 0.25 M NaCl, when a close similarity with the pattern obtained at low ionic strength is finally observed (Fig. 2(2)b).

These results reinforce the interpretation of the development of events during octamer reconstitution. They suggest that, with both 601 and the mutant sequences, H3/H4 are the first histones involved in nucleosome formation, interacting essentially (and asymmetrically with a penchant at the 601 sequence for the 5′ end) at the dyad axis but need to be assisted by H2A and H2B, even if these latter were not observed by us to interact alone with the DNA. In addition when we compare Figs 4 and 5 with the 601 sequence we clearly see that the influence of H2A/H2B on the Y-Y dimer signal is concentrated between SHL −3.5 and −2.5 and also on the 3′ side but to less extent. This would confirm that H2A/H2B is assisting the tetramer to bind to the DNA only subsequent to events creating the signal coming exclusively from the dimer/DNA interaction that appears at SHL −7 and −3.5 and same on the other side. This therefore confirms that the dimer interacts first with the tetramer helping it to bind and interact and then the dimer interacts directly with the DNA. In addition the binding of the tetramer alone or the tetramer and dimer at the centre of the 601 sequence must bend the DNA so the dimer can grab the DNA and fix it to stabilise the final nucleosome structure.

## Discussion

We have used UV laser induced PhAST of DNA to study nucleosomes formed with two DNA fragments, the 601 and mutant 601.2.4 sequences, which differ by only 15 base pairs concentrated in the 5′ left hand side of the dyad axis. Nucleosome reconstitution using octamers or (H3/H4)_2_ produced complexes having the expected MNase footprint on the 601 and mutant fragments centred on the dyad axis (Supplementary Fig. 3).

The photo-reactivity patterns seen following laser UV irradiation of the DNA alone or after reconstitution with the octamer (Fig. 1) concern all pyrimidine-pyrimidine steps (70 in the 601 sequence and 72 in the mutant sequence) and provide rich information on distortions and histone binding along the DNA sequences. As shown in Fig. 3, there is a clear correlation between roll angles in the nucleosome and changes in the probability of Y-Y dimer formation.

The numerous changes observed along the two sequences as exemplified by Fig. 2a and their interpretation in terms of rolls are meaningful for the mechanism underlying nucleosome formation. The implication is that most rolls in free DNA are not suitable to nucleosome formation. Since roll, twist and slide are globally coupled in free and bound DNA comprising nucleosomes^4^,^16^,^32^ it can reasonably be inferred that these characteristics of dinucleotides in free 601 and mutant sequences are not paramount in aiding DNA readout and increasing histone affinity. This assumption, already formulated from the examination of an ensemble of crystallographic nucleosome structures^33^ is also compatible with a recent experimental study of free DNAs related to the 601 sequence supporting the notion that nucleosome formation is more favoured by pre-adapted minor grooves than by parameters such as roll or twist^20^.

The reconstruction with (H3/H4)_2_ produces photo-reactivity patterns (Fig. 2b) in the central DNA region that correspond well with the interface between these histones and DNA (Fig. 2). With the 601 sequence, for the first time, changes are also observed throughout the 5′ half including regions that do not interact with H3 and H4 in the complete nucleosome (Fig. 2). However, these distortions are not identical to those observed in the nucleosome. The 5′ half of the 601 sequence is hence especially attractive for H3/H4, likely with several possible secondary binding sites. Overall, the analysis of nucleosome reconstructions using octamers and (H3/H4)_2_ shows that the interactions are weakened by mutations introduced in the 5′ half of the 601 sequence.

Changes in DNA structure during nucleosome formation were monitored as a function of the ionic strength (Figs 4 and 5); this was possible because of the unique advantages of the PhAST approach. Using the octamer and the 601 sequence at 1.5 and 1.0 M NaCl, the changes immediately around the dyad axis are the signature of initial binding of (H3/H4)_2_, in line with earlier experiments^34^,^35^ that established that (H3/H4)2 initiates nucleosome formation. However, the asymmetry in changes in intensities shows that the strongest interactions occur immediately 5′ of the dyad axis in agreement with other studies^19^. This asymmetry is not due to some interference with H2A and H2B, since it is also clearly observable during the reconstitution with only H3/H4.

Several studies suggested that electrostatic components, especially at SHL ± 1.5, are fundamental for nucleosome formation^28^,^36^,^37^. However, at 1.5 M NaCl, the electrostatic contacts and consequently the related DNA-histone interactions would be expected to be strongly weakened^37^. That robust interactions occur at high ionic strength was previously discussed in the context of a salt-induced DNA-histone dissociation study showing that salt stability increased with the G:C content^21^. However, at this point we would like to develop the idea that the nature of contacts made during indirect readout is not purely electrostatic but has a strong hydrophobic component. Hydrophobic interactions involving deoxyribose moieties were previously examined on a large dataset of DNA-protein complexes^38^ and explicitly mentioned in studies of crystallographic DNA-histone interfaces^9^,^10^,^11^,^28^. Our analysis of DNA-protein interfaces of five nucleosome structures (see Table 1 and Material and Methods for more details) shows that the number of DNA residues (in fact, mainly sugars) involved in hydrophobic interactions is larger with H3 and H4 than with H2A and H2B, and, importantly, independent of the DNA sequence. We would therefore suggest that the substantial hydrophobic component of the interface involving (H3/H4)_2_, complements the weakened electrostatic action and plays a role in anchoring of (H3/H4)_2_ at high ionic strength, without excluding other potential factors such as ionic contacts.

Coming back to the asymmetric behaviour of the 5′ and 3′ sides of the dyad, we propose that the 5′ side of the dyad of the 601 sequence takes advantage of the favourable periodicity in flexibility^39^ and preformed groove shape^20^. At the 3′ side of the dyad, these parameters are weaker^20^,^39^ and, to compensate this deficit, the complex requires lower ionic strength (1.0 M) to be stabilized. More specifically, the differential behaviour of the 5′ and 3′ sides of the 601 sequence could also be related to TpA steps, deemed to easily accommodate histone-induced DNA distortions. Indeed, the 5′ side contains four TpA that coincide with points of maximal pressure in the nucleosome while the 3′ side includes only one TpA^10^,^21^.

The situation with the mutant sequence (Fig. 4(2)) can be described by a similar logic. Here in the presence of the octamer or (H3/H4)_2_ the pattern on both sides of the dyad is quite symmetrical at 1.5 and 1.0 M NaCl, with major changes occurring only at 1.0 M (Figs 4(2) and 5(2)). The 5′-side is clearly less efficient for recruiting H3/H4 in the mutant sequence than in the 601 sequence. Only six base pairs differ between SHL-3 and SHL-1.0 (CTAGCACCGCTTAAACGCAC in 601, CTGGATCCGCTTGATCGAAC in the mutant), which alter in particular the TTAAA element (underlined), a strong point for anchoring H3/H4^10^,^25^,^30^,^34^. In the mutant region, the favourable structural properties^20^,^37^ of the free TTAAA element are weakened and, in addition, the alternation of flexible and stiff elements detectable in the 601 sequence is lost. Such effects would be sufficient to affect the ease of recognition.

Importantly, the favourable characteristics of the 5′ side of the 601 sequence seem to enhance interactions involving the 3′ side, typical of a cooperative process. When these characteristics are modified, as in the 5′ side of the mutant sequence, the interactions between the 3′ side and H3/H4 are also compromised (Fig. 4(2)). This scenario is consistent with results obtained with the 601 and mutant sequences in the presence of only (H3/H4)_2_ (Fig. 2(1)b,(2)b), with changes in intensities much higher for the 601 sequence than for the mutant sequence.

A study of free energies measured with histone octamer and (H3/H4)_2_ tetramer concluded that interactions with (H3/H4)_2_ dominate nucleosome positioning^8^. The same study highlighted the remarkable conservation of the DNA region involved in the H3/H4 contacts in artificial sequences selected for their maximal ability to form nucleosomes. Here, instead of two independent experiments, our approach allows to simultaneously demonstrate that the first stage of reconstitution involves recognition by (H3/H4)_2_ and is strongly modulated by the DNA sequence. We also suggest that a series of cooperative interactions, during and after these initial interactions, allows successive binding and bending of the DNA. Our results also help to precise the notion of dyad axis. The dyad axis location initially derives from the crystallographic structures, compatible with the centre of the “footprint” of the octamer. However we believe that this location is simply a consequence of H3/H4 binding and is not *per se* a determining factor.

The results described in the present work suggest a simple general model for nucleosome formation. An initial readout mechanism involves narrow minor grooves that interact with positive arginine side chains of (H3/H4)_2_ due not only to electrostatic contacts but also to substantial hydrophobic contacts between the aliphatic side chains of amino acids and DNA carbon atoms in sugars and bases. An initial complex is thus stabilized in which narrow minor grooves are already oriented towards the (H3/H4)_2_ core. This binding by (H3/H4)_2_ induces changes, in a word bending, of the DNA; the formation of this first complex is extremely sensitive to the DNA sequence and ultimately defines the position of the final dyad axis. The final complex is obtained through the binding of H2A and H2B, which seems to be less dependent on the DNA sequence and is guided by interactions with (H3/H4)_2_ already bound to the DNA, and distortions induced in the DNA by (H3/H4)_2_. Small rearrangements notably at the ends of the DNA sequence then lead to a final stable complex.

In conclusion we believe that positional sequences *per se* probably no longer exist in their original form since their role has essentially been superseded by the need to regulate by repositioning using remodelling mechanisms. However we suggest that residual positional signals persist in regions involved in the crucial first step corresponding to the (H3/H4)_2_ recruitment. Thus, the interpretation of large data from extensive mappings of nucleosome positioning would need to focus on windows of 70 bp, approximately the DNA length covered by (H3/H4)_2_ rather than the whole length of nucleosome DNA, incorporating the periodicity of intrinsic DNA flexibility at the dinucleotide level with respect to structural changes that promote or are compatible to bends towards the minor groove. Indeed in Caserta *et al*.^40^ a 51 bp window was used. We note that *in vivo* if the DNA sequence in the (H3/H4)_2_ binding region is insufficient for positioning this can, in principle, be compensated by rotational sequence determinants in the H2A/H2B binding regions. The latter would increase affinity. Such an effect would be consistent with the previously reported patterns of nucleosomal DNA sequence periodicities^40^,^41^,^42^. We further note that these parameters become subordinate to other criteria in subsequent rearrangements of nucleosome positioning carried out by extraneous factors such as remodelers, transcription/replication machinery and so forth, but that these parameters still play a major role in moderating such effectors.

Finally the PhAST approach outlined here describes a novel, precise and subtle technique that introduces new elements into the current paradigm for the definition of nucleosome positioning and suggests new means of studying and understanding chromatin organisation at a dynamic and molecular level.

## Material and Methods

### DNA Preparation

Plasmids containing a 601 fragment (pGEM3Z-601) were kind gifts from Dr. David Bensimon at the Ecole Normale Supérieure de Paris. To prepare linearized DNA, the plasmid was digested with PvuII (New England BioLabs), which digests both ends of the 601 sequence (Supplementary Fig. 2a), and then purified by phenol-chloroform extraction and ethanol precipitation. A similar process was carried out for the 601.2.4 sequence (Supplementary Fig. 2b). Both sequences are shown in Supplementary Fig. 2c.

### Nucleosome reconstitution

Nucleosomes were reconstituted with the salt dilution protocol provided by New England BioLabs with a slight modification; Human recombinant histone H2A/H2B dimer (1.5 μg) and/or histone (H3/H4)_2_ tetramer (1.5 μg) (New England BioLabs) were mixed with the linearized DNA (3 μg) in 10 μl of 2 M NaCl, The mixture was incubated at room temperature for 30 min before the salt concentration was lowered to 100 mM by adding dilution buffer (10 mM Tris-Cl, pH 7.5, 1 mM EDTA, 0.05% NP-40, 5 mM 2-mercaptoethanol, 0.1 mM PMSF) five times every 20 min. Nucleosome solutions (DNA concentration, 10 ng/μl) were concentrated to ~50 ng/μl of DNA concentration using a MultiScreen Ultracel-10 filter plate (Millipore).

### Partial MNase digestion

25 μl of reconstituted nucleosomes or DNA alone were digested with 1 mU/ml of MNase from *Staphylococcus aureus* (Sigma-Aldrich) for 15 min at 37 °C in the presence of 5 mM CaCl_2_. To stop the reaction, 2.5 μl of 500 mM EDTA was added and the DNA was purified by phenol-chloroform extraction and ethanol precipitation.

### Photochemical Analysis of Structural Transitions (PhAST)

20 μl of the reconstituted nucleosomes or DNA alone (DNA concentration, ~50 ng/μl) were placed in 0.5 ml Eppendorf tubes and irradiated with 5-ns-long pulses of 266 nm UV laser beam at a frequency of 10 Hz for a period of 1 sec as described in^12^ After irradiation, DNA fragments were purified by phenol-chloroform extraction and ethanol precipitation.

### Primer extension and capillary electrophoresis

The sites of the UV photoproducts and the MNase digestion were analyzed by single-cycle primer extension using Taq DNA polymerase (New England BioLabs). Two primers (Primer A: 5′-GCTATGACCATGATTACGCCAAGC-3′, Primer B: 5′- AGGGTTTTCCCAGTCACGACGTT-3′) with 6-FAM labelling at 5′ end were used for the UV irradiated samples to analyze both strands of the 601 and 601.2.4 sequences. For the samples digested by MNase, the labeled primer A and primer B was used for the 601 and 601.2.4 sequences, respectively. Primer positions are shown in Supplementary Fig. 2. For the extension, 200–500 ng of the UV irradiated or the MNase digested DNA were added with 20 μl of final volume of an amplification mixture containing 0.2 mM of each dNTP, 0.2 μM of the end-labeled primer, and 0.025 units/μl of Taq polymerase in Taq standard buffer (New England BioLabs). The samples were then denatured for 5 min at 95 °C and subjected to extension (1 min at 55 °C for primer annealing and 8 min at 72 °C for extension) in a thermal cycler. After the primer extension reaction, the products were collected by ethanol precipitation. The samples were re-suspended in 10 μl of deionized formamide containing 0.25 μl of the GeneScan-600 LIZ internal size standard (Applied Biosystems) and separated by a capillary electrophoresis instrument (3500 genetic analyser, Applied Biosystems).

### Analysis of DNA-protein interface

The DNA-protein interface of nucleosome structures were analysed with PDidb^43^ that allows the identification of DNA and protein residues in contact and to precise the type of contact, hydrogen bond, ionic bond, hydrophobic interaction or simple proximity between atoms. This analysis was carried out using a drastic distance cut-off of 4 Å. In other words, two residues are considered in contact if they are distant of 4 Å or less. In practice this cut-off eliminates potential water mediated hydrogen bonds, which cannot be ascertained in X-ray structures with restricted number of resolved water molecules. Such analysis therefore reports only the closest contacts between DNA and proteins. Five X-ray structures of nucleosome were considered; their PDB codes are 3MVD, 3LZ0, 3LZ1, 3UT9 and 1KX3. 3MVD, 3LZ0 and 3LZ1 contain the 601 sequence and 3UT9 is formed with the 601L sequence, the palindromic derivative of the 5′part of the 601 sequence. 1KX3 contains the human a-satellite sequence that largely differs from the 601 sequence (49% of identity). This structure enabled to ensure that the DNA-protein contacts do not crucially depend on the DNA sequence. Indeed, the analysis showed a remarkable coherence between the contacts observed in the five structures (Table 1).

We would like to point out that substantial parts of histone tails are not resolved in these structures. Hence, the interface analysis only accounts for the contacts between the DNA and the structured histone core, which are yet largely dominant in the interface constitution^11^.

## Additional Information

**How to cite this article**: Hatakeyama, A. *et al*. High-resolution biophysical analysis of the dynamics of nucleosome formation. *Sci. Rep.* **6**, 27337; doi: 10.1038/srep27337 (2016).

## Supplementary Material

Supplementary Information

## Figures and Tables

**Table 1:** This table summarizes the interface analysis of five X-ray structures of nucleosome referred by their PDB codes.

		3MVD	3LZ0	3LZ1	3UT9	1KX3	N_AV_	SD(N_AV_)
Contacts with (H3)_2_	N_Total_	45	44	41	44	37	42.2	3.3
	N_hydrophobic_	16	19	20	15	19	17.8	2.2
	% N_hydrophobic_	**0.36**	**0.43**	**0.49**	**0.34**	**0.51**	**0.4**	**0.1**
Contacts with (H4)_2_	N_Total_	14	17	15	18	13	15.4	2.1
	N_hydrophobic_	7	6	3	8	8	6.4	2.1
	% N_hydrophobic_	**0.50**	**0.35**	**0.20**	**0.44**	**0.61**	**0.4**	**0.2**
Contacts with (H2A)_2_	N_Total_	30	28	29	26	21	26.8	3.6
	N_hydrophobic_	8	6	6	6	6	6.4	0.9
	% N_hydrophobic_	0.27	0.21	0.21	0.23	0.28	0.2	0.1
Contacts with (H2B)_2_	N_Total_	25	22	22	24	19	22.4	2.3
	N_hydrophobic_	7	2	4	6	7	5.2	2.2
	% N_hydrophobic_	0.28	0.09	0.18	0.25	0.37	0.2	0.1

It reports the number of DNA residues involved in DNA-protein contacts, regardless of the type of contact (N_total_), and the number of DNA residues specifically engaged in hydrophobic contacts (N_hydrophobic_). %N_hydrophobic_ is the percentage of DNA residues specifically engaged in hydrophobic contacts (N_hydrophobic_/N_total_). The last two columns give the average values and standard deviations of N_total_, N_hydrophobic_ and %N_hydrophobic_.
