# Supplementary material for: High-resolution biophysical analysis of the dynamics of nucleosome formation
Source: Sci Rep. 2016 Jun 6;6:27337. doi: 10.1038/srep27337 (PMC4897087; doi:10.1038/srep27337)
Supplement: Supplementary Information [file srep27337-s1.pdf]

High-resolution biophysical analysis of the dynamics of nucleosome formation.

Akiko Hatakeyama[1], Brigitte Hartmann[1], Andrew Travers[2,3,] Claude Nogues [1]and Malcolm Buckle [1*].

[1]. LBPA, IDA, ENS Cachan, CNRS, Université Paris-Saclay, F-94235, Cachan, France

[2]. MRC Laboratory of Molecular Biology, Francis Crick Avenue, Cambridge Biomedical Campus Cambridge, CB2 0QH UK

[3]. Department of Biochemistry, University of Cambridge, Tennis Court Road, Cambridge, CB2 1GA, UK

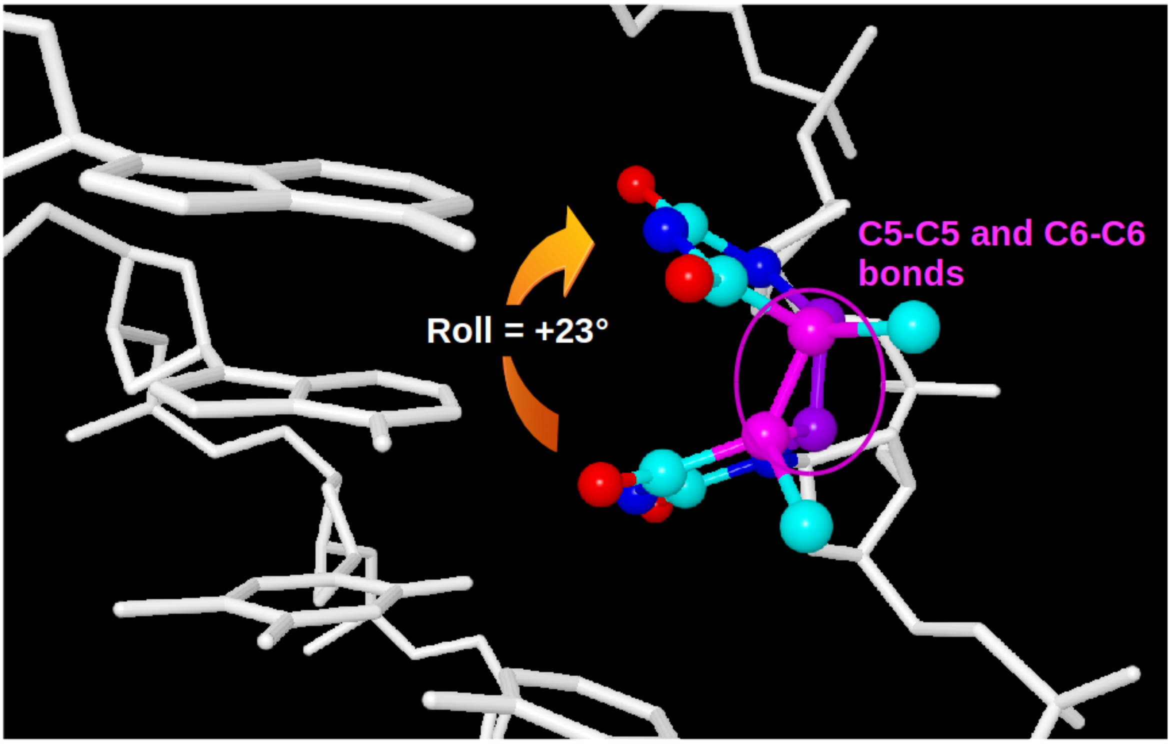

Supplementary Figure 1. Lateral view of TT dimer.
The crystallographic structure of a DNA decamer containing a thymine-thymine dimer [15] (PDB code 1SM5) shows that the formation of C5-C5 and C6-C6 covalent bonds (magenta) between two successive thymines induces a strong positive roll.

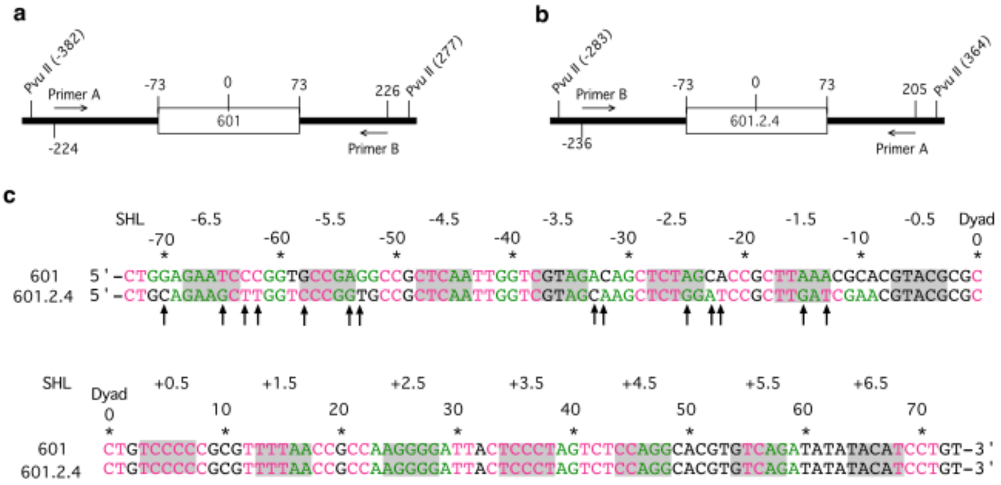

Supplementary Figure 2. Diagram of DNA fragments containing the (a) 601, (b) 601.2.4 elements and (c) the 5' half and 3' half of DNA sequence of the 601 and 601.2.4 elements; the pyrimidine-pyrimidine steps are pink and purine-purine steps (corresponding to pyrimidine-pyrimidine steps on the opposite strand) are green. Arrows indicate mutation sites in the 601.2.4 sequence.

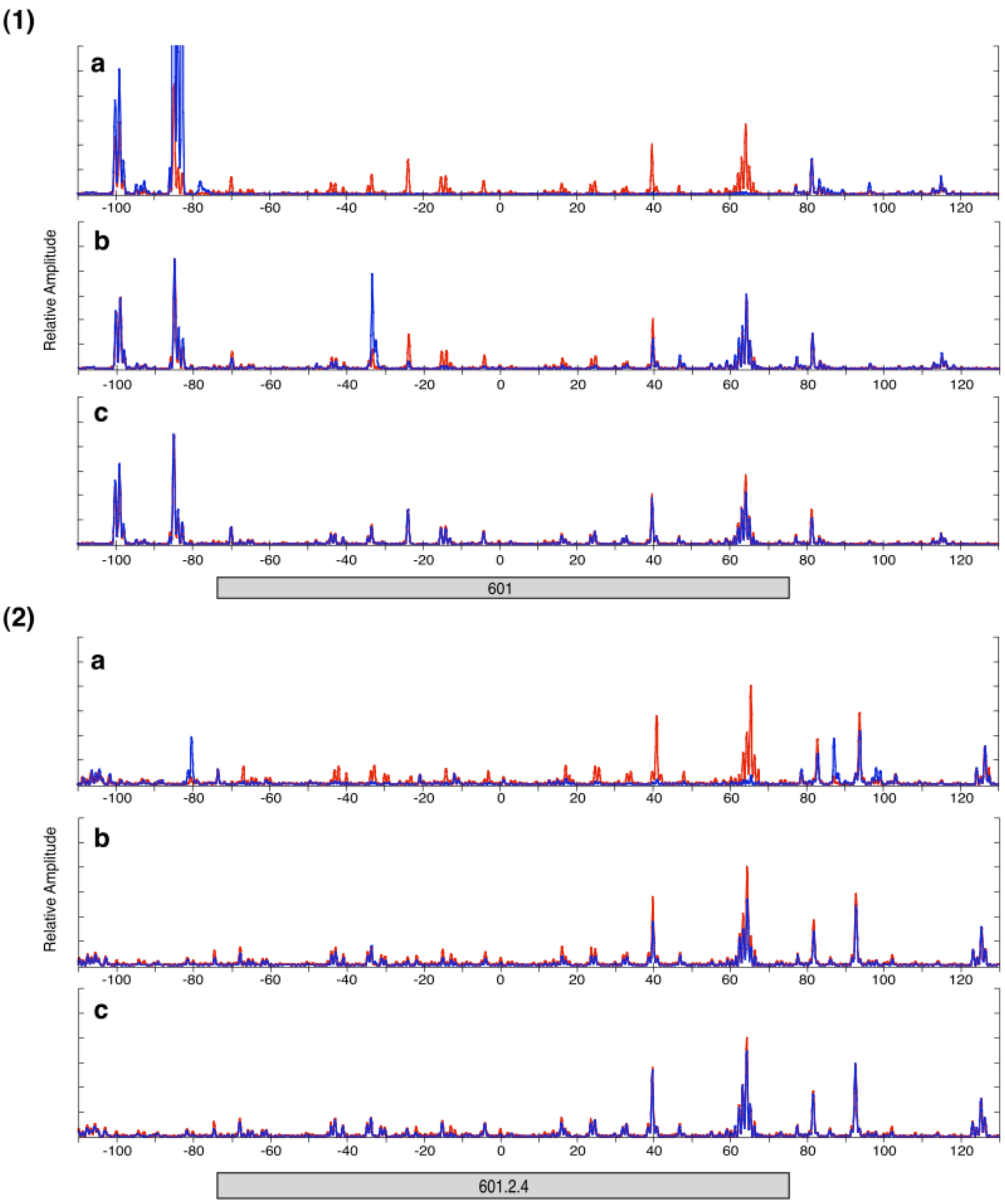

Supplementary Figure 3.

Primer extension then capillary electrophoresis after MNase digests of

1) 601 fragment (a) histone $(H3/H4)_2$ tetramer and H2A/H2B dimers, (b) only $(H3/H4)_2$, and (c) only H2A/H2B dimers. 2) 601.2.4 fragments (a) histone $(H3/H4)_2$ tetramer and H2A/H2B dimers, (b) only $(H3/H4)_2$ tetramer, and (c) only H2A/H2B dimers. Red lines show naked DNA, blue lines show reconstituted DNA.
2) 601.2.4 fragment fragment (a) histone $(H3/H4)_2$ tetramer and H2A/H2B dimers, (b) only $(H3/H4)_2$, and (c) only H2A/H2B dimers. 2) 601.2.4 fragments (a) histone $(H3/H4)_2$ tetramer and H2A/H2B dimers, (b) only $(H3/H4)_2$ tetramer, and (c) only H2A/H2B dimers. Red lines show naked DNA, blue lines show reconstituted DNA.

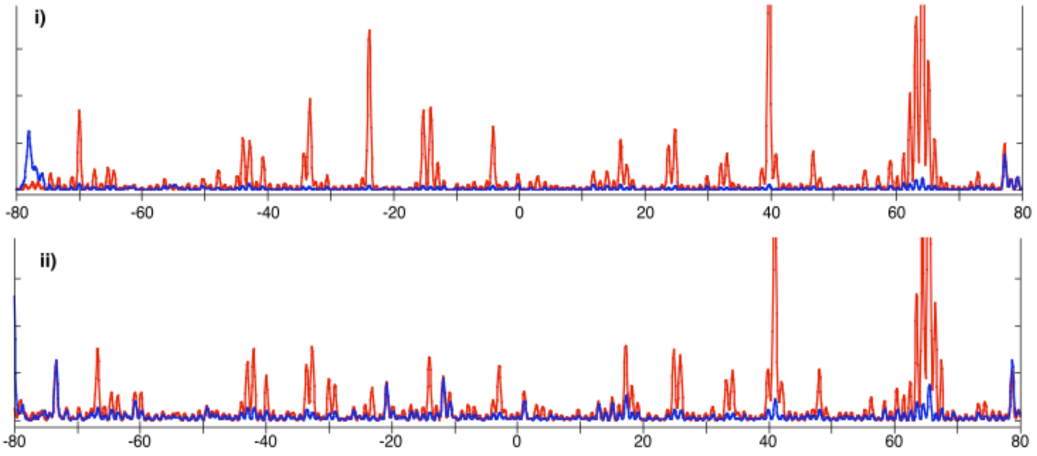

Supplementary Figure 4.

Enlarged central region of footprints shown in supplementary figure 3.

i) 601 fragment ii) 601.2.4 fragments. Red lines show naked DNA, blue lines show reconstituted DNA.
